# Establishing Accelerometer Cut-Points to Classify Walking Speed in People Post Stroke

**DOI:** 10.3390/s22114080

**Published:** 2022-05-27

**Authors:** David Moulaee Conradsson, Lucian John-Ross Bezuidenhout

**Affiliations:** 1 Division of Physiotherapy, Department of Neurobiology, Care Sciences and Society, Karolinska Institutet, 141 83 Stockholm, Sweden; david.m.conradsson@ki.se; 2 Women’s Health and Allied Health Professionals Theme, Medical Unit Occupational Therapy and Physiotherapy, Karolinska University Hospital, 171 64 Stockholm, Sweden; 3 Faculty of Community and Health Sciences, University of Western Cape, Cape Town 7535, South Africa

**Keywords:** accelerometers, ActiGraph, gait speed, objective measurement, ROC analysis, stroke, wearable sensors

## Abstract

While accelerometers could be used to monitor important domains of walking in daily living (e.g., walking speed), the interpretation of accelerometer data often relies on validation studies performed with healthy participants. The aim of this study was to develop cut-points for waist- and ankle-worn accelerometers to differentiate non-ambulation from walking and different walking speeds in people post stroke. Forty-two post-stroke persons wore waist and ankle accelerometers (ActiGraph GT3x+, AG) while performing three non-ambulation activities (i.e., sitting, setting the table and washing dishes) and while walking in self-selected and brisk speeds. Receiver operating characteristic (ROC) curve analysis was used to define AG cut-points for non-ambulation and different walking speeds (0.41–0.8 m/s, 0.81–1.2 m/s and >1.2 m/s) by considering sensor placement, axis, filter setting and epoch length. Optimal data input and sensor placements for measuring walking were a vector magnitude at 15 s epochs for waist- and ankle-worn AG accelerometers, respectively. Across all speed categories, cut-point classification accuracy was good-to-excellent for the ankle-worn AG accelerometer and fair-to-excellent for the waist-worn AG accelerometer, except for between 0.81 and 1.2 m/s. These cut-points can be used for investigating the link between walking and health outcomes in people post stroke.

## 1. Introduction

Walking is the most common form of physical activity (PA) [1], and how fast a person walks (i.e., gait speed) is associated with health, risk of disease, and mortality in the future [2,3,4]. Recovering walking ability is an important goal for people post stroke [5] and a useful marker of independence, functioning and health status across the continuum of recovery after stroke [6,7,8]. In stroke research and clinical practice, gait speed is commonly assessed in a standardized setting using a performance-based test (e.g., 10 m walk test or 6 min walk test) [8]. Although the clinical assessment of gait speed provides important information of the level of function post stroke, it does not provide quantitative information of actual walking ability outside the clinical setting (e.g., home environment).

The ActiGraph GT3X+ (AG; ActiGraph Corp., Pensacola, FL, USA) is a small triaxial accelerometer (dimensions: 4.6 × 3.3 × 1.5 cm; weight: 19 g) with a dynamic range of ±6 G (1 G = 9.81 m/s^2^) [9], and it is one of the most used accelerometers for measuring PA [10,11,12]. The AG device measures continuous acceleration signals related to PA that are in turn converted to a ‘count’, which is defined as the acceleration signal crossing a proprietary amplitude threshold (greater counts indicating more intense PA). Population-specific count cut-points have been developed to distinguish between different PA levels (i.e., sedentary, light-intensity PA, and moderate-to-vigorous PA) [13]. The AG device can be worn on different body positions (e.g., waist and ankle) and can continuously measure PA over a prolonged period (up to six weeks at 30 Hz). Although previous studies have mostly used energy-expenditure measurements (such as indirect calorimetry) as the criterion measure [14,15,16], accelerometers measure a biomechanical characteristic of PA, so researchers have suggested that PA intensity can be interpreted in the biomechanical domain, e.g., by referring the AG output (i.e., counts) to walking speed categories [17].

Previous studies have demonstrated that the AG device underestimates energy expenditure in people post stroke [18,19,20]. This is believed to be influenced by the often-altered gait pattern post stroke (e.g., hemiparesis) [21] that, combined with slow walking speed, leads to lower AG output even if a fairly high energy expenditure is required for ambulation [20,22]. Slow walking speed also affects the AG accelerometer’s step detection accuracy, and previous studies have demonstrated the poor accuracy of the AG device when detecting steps in people post stroke—especially when worn on the waist and during slow walking (<0.6 m/s) [23,24]. Furthermore, the authors of most previous studies developed accelerometer cut-points using epoch lengths of 60 s [12,25]. However, such cut-points are difficult to apply to populations with impaired gait (e.g., following stroke) who rarely perform steady-state walking bouts for ≥60 s [26]. AG cut-points reflecting shorter epoch length (e.g., 15 s) might therefore be better suited to measure frequent short-duration walking bouts with low numbers of sequential steps for people post stroke.

Currently, there are no AG cut-points for measuring the gait speed developed for people post stroke. Importantly, the assessment and classification of walking speeds in daily life requires not only cut-points to separate different walking speeds but also a minimum cut-point to separate non-walking from walking. The aim of this study was therefore to develop cut-points for waist- and ankle-worn AG accelerometers to differentiate non-ambulation from walking and different walking speeds in people post stroke. Since previous studies have shown that the accuracy of AG accelerometers is affected by placement on the body (e.g., waist or ankle) [23,24], the axis analyzed, and data-filtering options [11], these parameters should also be taken into consideration while developing cut-points.

## 2. Materials and Methods

### 2.1. Study Participants

People who had a stroke ≥3 months prior to study participation and had the ability to ambulate with/without a walking device for 2 min were recruited from two rehabilitation centers in Stockholm, Sweden, through advertisement platforms. Exclusion criteria were cognitive deficit, severe neglect and aphasia affecting the ability to give written consent and follow instructions. The project was approved by the Regional Board of Ethics in Stockholm (2017/1626-31 and 2018/2524-32), and all study participants gave written consent prior to study participation.

### 2.2. Data Collection

All participants attended one session at a rehabilitation facility that included: (a) structured interviews of demographics and personal factors; (b) clinical tests and questionnaires regarding the severity of stroke symptoms, cognition, activities in daily living, and functional mobility; and (c) the assessment of walking and non-ambulation activities using AG accelerometers. All assessments were conducted by a physiotherapist with experience in working with people post stroke. 

Demographic data included sex, age, living situation, height, weight, employment status, mobility status (unaided/walking aid), and information about stroke (i.e., years since stroke, type of stroke and affected side). The severity of stroke and cognition was assessed with the National Institutes of Health Stroke Scale (NIHSS) [27] and Montreal Cognitive Assessment (MoCA) [28], respectively. Additionally, personal activities of daily living (e.g., bathing, dressing and toileting) and instrumental activities of daily living (e.g., shopping, cleaning and transportation) were assessed using the Katz ADL Index Extended (KATZ Index) [29].

**Figure 1:**
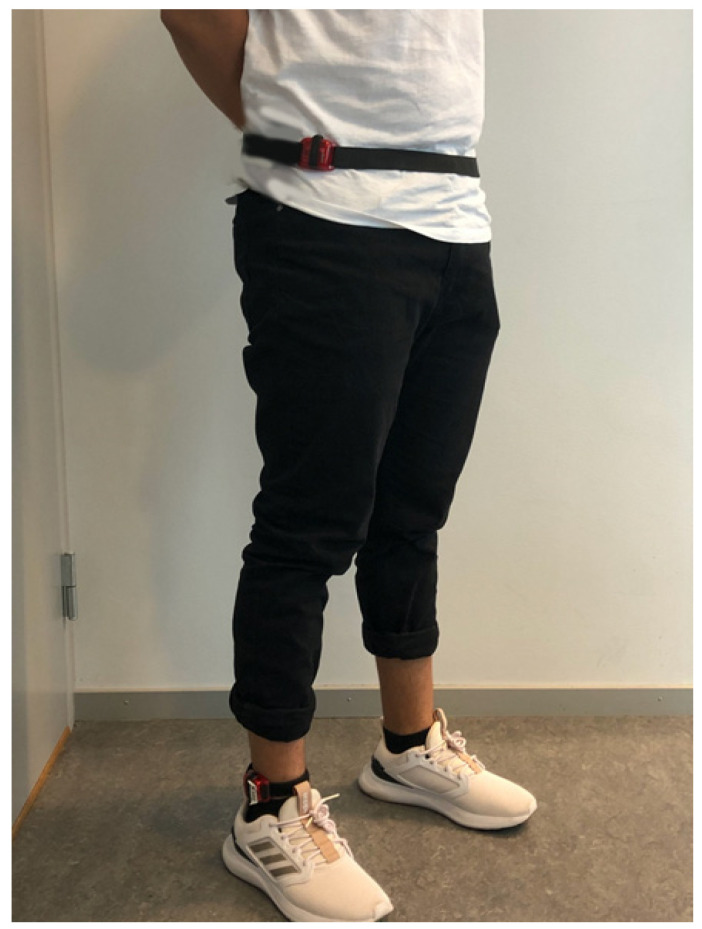
ActiGraph positioned around the right waist (above the iliac crest) and right ankle (proximal to the lateral malleolus).

Participants performed three non-ambulation activities and two different walking tests (see Table 1). The non-ambulation activities were chosen due to their occurrence in daily living, and the duration was chosen to reach steady state. For the walking activities, the 6-minute walk test was used since it is an established clinical assessment of functional ability [30]. Prior to the assessment of these activities, participants were fitted with two AG accelerometers (model GT3X+, ActiGraph Corp., Pensacola, FL, USA). The position of the accelerometers was guided by the work of Webber and St. John [31], with the AG accelerometers attached around the right waist (above the iliac crest) and ankle (proximal to the lateral malleolus) on the side of the body not affected by the stroke symptoms (see Figure 1). These placements of AG accelerometers have been the most commonly used in previous studies of PA in people post stroke [23,32,33]. The AG devices recorded time-series acceleration data in three axes at a sampling rate of 30 Hz [9]. During the assessment of non-ambulation activities and walking, the test leader noted the start and stop times to extract the corresponding data from the AG sensor signal. During the assessment of walking, the test leader recorded the distance for the self-selected and brisk walking trials.

### 2.3. Data Management and Analysis

ActiGraphs’ corresponding software (ActiLife^®^, version 6.13.4, ActiGraph Corp., Pensacola, FL, USA) was used to download and manage the AG waist and ankle data. To extract PA movements and attenuate artifacts, ActiLife software uses a bandpass filter between 0.25 and 2.5 Hz [35]. The raw acceleration signal (positive and negative) is digitized by a 12-bit analog-to-digital converter, which corresponds to 4096 levels of acceleration measurements (i.e., 2^12^ = 4096), where zero acceleration (i.e., no movement) is associated with the center of the analog-to-digital scale (i.e., 4096/2 = 2048). The acceleration in both positive and negative directions, which is proportional to the vector component of the acceleration, deviates the signal from the zero acceleration. The acceleration signal is then converted to counts, where a count is defined as the acceleration signal crossing a ActiLife proprietary amplitude threshold. The AG count data for three axes (vertical, anterior–posterior, and medio-lateral) and vector magnitude (i.e., summation of counts for all three axes, VM) were then summed to 15 s and 1 min epochs and exported as a MATLAB file using ActiLife. The 15 s and 1 min epochs were chosen to account for both frequent short- and long-duration walking bouts occurring in daily living. The AG data were obtained using both the default filter (0.25–2.5 Hz) and the low-frequency extension filter [36,37]. The latter is recommended for low-intensity activities [38], such as slow walking [34].

The AG data were segmented into the individual activities (i.e., the total duration of each walking or non-ambulatory activity) using the start and stop times recorded by the test leader. The first and last 15 s of each activity were disregarded since they comprised the initialization and termination of the activities and to ensure that steady-state movements were reached. All the non-ambulation activities (tasks 1–3) were pooled together to form one non-ambulatory category.

For the walking conditions, the speed of each trial was calculated by dividing the distance walked by the time recorded to cover the distance, and all trials were assigned to one of the following speed categories: 0.4–0.8 m/s, 0.81–1.2 m/s, and >1.2 m/s. These speed categories were selected because they are often used in stroke research to discriminate between limited community ambulators (0.4–0.8 m/s) and unlimited community ambulators (0.81–1.2 m/s) [39]. 

The vertical count of the waist placement (default filtering) is the most used variable of AG sensors to measure PA [10], while ankle-worn sensors utilizing the low-frequency extension filter have presented improved step detection during slow walking [40]. Therefore, the cut-points were developed for both sensor placement and filtering settings in the *y*-axis and VM. Receiver operating characteristic (ROC) curve analysis was used to develop the cut-points for the ankle- and waist-worn AG sensors to distinguish non-ambulatory activities from walking and between different group speeds. The cut-point was defined as the point on the ROC curve that maximized the sensitivity and specificity, i.e., the point nearest to sensitivity = 1 and 1-specificity = 0 (the point closest to the upper left corner of the ROC curve) [41,42]. A test of perfect classification has sensitivity = 1, 1-specificity = 0, and AUC = 1. The area under the curve (AUC) was used to provide an empirical basis for determining the most appropriate cut-points, where an AUC < 0.7 = poor; 0.7–0.79 = fair; 0.8–0.89 = good; ≥0.90 = excellent; and 1 = perfect test [42,43]. Descriptive statistics were used to describe the stroke participant and AG data. 

## 3. Results

### 3.1. Study Participants

Forty-two people post stroke participated in this study (see Table 2 for participants’ characteristics). There was an almost even distribution between women (55%) and men (45%), and most participants had experienced an ischemic stroke (74%) and were independent in primary ADL (87%) but not in instrumental ADL (48%). Twelve (29%) participants used a walking device (walking cane: *n* = 9; stroller: *n* = 3) while performing the walking tests. Five participants were excluded due to failure to complete the 6 minutes of walking. Due to sensor availability, 11 participants only used the waist AG accelerometer. The speed classification along with the median counts per 15 s and 1 min epochs are presented in Table 3.

### 3.2. Cut-Points

**Figure 2:**
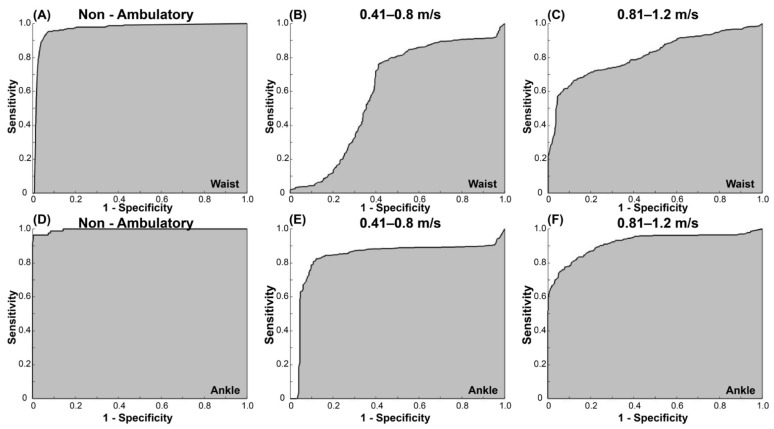
ROC curves for the VM counts/15 s for the waist-worn AG accelerometer for (**A**) non-ambulation, (**B**) 0.41–0.8 m/s, and (**C**) 0.81–1.2 m/s; ROC curves for the VM counts/15 s for the ankle-worn AG accelerometer for (**D**) non-ambulation, (**E**) 0.41–0.8 m/s, and (**F**) 0.81–1.2 m/s.

Table 4 shows the sensitivity and specificity for each speed category for the waist- and ankle-worn AG accelerometers using the vertical and VM counts for both 15 s and 1 min epochs. Across all categories and regardless of the epoch length, the AUC for the waist-worn AG accelerometer was poor-to-excellent (0.60–0.91) for both vertical and VM counts, with the non-ambulatory category having the highest sensitivity and specificity (Table 4). For the ankle-worn AG accelerometer, the AUC ranged from fair to excellent (0.76–0.99) for both vertical and VM counts and epoch lengths. Table 5 shows the optimal recommended cut-points for the waist- and ankle-worn AG accelerometers based on 15 s epochs. For the waist-worn AG accelerometer, classification accuracy was excellent for the non-ambulation threshold (Figure 2A), poor for 0.41–0.8 m/s (Figure 2B), and fair for 0.81–1.2 m/s threshold (Figure 2C). For the ankle-worn AG accelerometer, classification accuracy was good-to-excellent for all categories (Figure 2D–F). The results from the low-frequency extension filter did not improve the accuracy of the classification. 

## 4. Discussion

To our knowledge, this is the first study to provide AG cut-points for measuring walking speeds in people post stroke. The results showed the VM counts using a 15 s epoch length for both the waist- and ankle-worn AG accelerometers to be most sensitive in differentiating between non-ambulatory and walking and in classifying different walking speed categories in people post stroke. Our findings also showed that the ankle-worn AG accelerometers were more sensitive in distinguishing between walking speeds compared to the waist-worn AG accelerometers, and the low-frequency extension filter did not improve the accuracy. The resultant cut-points may be used to quantify PA behavior in people post stroke and to ascertain regular free-living walking speed in this population.

The authors of previous studies have developed AG cut-points for walking conditions in healthy older adults with speeds between 0.7 and 1.3 m/s [42,44] and people with Parkinson’s disease at speeds between ≤1.04 and ≥1.31 [42], but no previous study has defined cut-points for slow walking among people post stroke. Slow walking speeds have been linked to dependency in daily living, declines in function, and health status after stroke [6,7,8]. It is therefore important to accurately detect walking speeds in daily life in order to identify individuals post stroke who have an increased risk of deteriorating health. Compared to the AG cut-points defined by Nero et al. 2015 for walking speeds of older people with Parkinson’s disease [42], the cut-points presented here for around 1.0 m/s are higher. Nero et al. 2015 reported a VM cut-point for a waist-worn AG accelerometer of 470 counts/15 s and 851 counts/15 s to separate walking at 1.04 and ≥1.31 m/s, respectively, whereas in our study, we reported between 573 and 990 counts/15 s to separate walking at > 0.81–1.20 m/s and ≥991 counts per 15 s to separate walking at ≥1.20 m/s. The difference in the developed cut-points could be attributed to the difference in gait-speed groups and/or the two populations having different gait patterns. People post stroke often present with an asymmetric gait pattern characterized by jerking movements of the trunk and a reduced coordination and range of motion of the affected leg [45]. Hemiparetic gait could in turn cause the movement of the body to induce high amplitudes of the accelerometer signal. In contrast, people with Parkinson’s disease often present with reduced speed, step length, and cadence during walking [46], which could reduce the accelerometer signal. Furthermore, the waist AG cut-point (≤140.4 vector magnitude counts/15 s) developed in the present study for differentiating slow walking (0.41–0.8 m/s) from sedentary behavior is similar to previous cut-points reported for older adults [25]. 

For people post stroke, ankle-worn AG accelerometers have been proposed as an alternative to the waist-worn AG accelerometers, which have been the most used in previous studies [12]. The ankle placement of the AG accelerometer compared to the waist has been shown to be more accurate for step detection in people post stroke [23] and in healthy individuals [40]. Our results are in line with these findings, with the present results showing the ankle-worn AG accelerometers to be more sensitive in distinguishing non-ambulation from walking and different walking speeds compared to the waist-worn AG accelerometers. The difference in accuracy could be attributed to the attenuation of the signal amplitude from the distal to proximal placement (i.e., higher signal amplitude at the ankle compared to the waist-worn AG accelerometer). This is especially evident during slow walking, where the acceleration signal at the waist-worn AG accelerometer is likely not sufficient to cross the proprietary AG software amplitude threshold to determine a count [40,47]. Although we consider using waist-worn AG accelerometers to measure PA in daily living to be more feasible compared to ankle-worn AG accelerometers, our results and previous findings suggest that ankle-worn AG accelerometers should be used for the more accurate analysis of walking in daily life.

The present results demonstrate an overall higher sensitivity in differentiating between non-ambulatory and walking and in classifying different walking speed using 15 s epochs compared to 1 min epochs (Table 3 and Table 4). In our study, we deliberately decided to use a shorter epoch length (15 s) since populations with impaired gait rarely perform steady-state walking in longer bouts. We also included the analysis of the longer epoch length (i.e., 1 min) because it has been documented that longer epoch often results in the improved accuracy of accelerometer cut-points when differentiating activity vs. sedentary behavior [25]. However, 1 min cut-points are difficult to apply to people with stroke who rarely walk in bouts of ≥60 s. AG cut-points reflecting a short epoch length might therefore be better suited to measure frequent short-duration walking bouts with low numbers of sequential steps for people post stroke [26]. Since accelerometer cut-points for a given epoch length and axis cannot simply be extrapolated to other epoch lengths or units [25], we recommend that the cut-points defined in our study are applied using 15 s epochs.

The low-frequency extension filter increases the sensitivity of the accelerometer signal during low-intensity movements and should theoretically be more sensitive in measuring slow walking speeds. However, the present results do not support an improved accuracy of classifying gait speed using the AG low-frequency-extension filter. Previous studies have reported the improved accuracy of detecting steps while walking using the AG low-frequency extension filter during slow walking in healthy individuals [40] but not in people post stroke [23]. As discussed above, the high amplitudes of the accelerometer signal despite slow walking are a plausible reason why this filter does not lead to a better classification of walking speed in people post stroke. It is also worth noting that 12 participants used a walking device during the walking test, though sub-analysis showed no difference in the sensitivity between the group using a walking device and the group not using a walking device.

There is a general shift from traditional cut-point (i.e., ROC analysis) development towards fusion algorithms (i.e., various filtering techniques) and machine learning approaches [48]. Such approaches could result in the better classification of different walking speeds in people post stroke. However, the implementation of these algorithms (e.g., machine learning) requires programming skills and specific software amongst health researchers and healthcare professionals that are sometimes absent in research or clinical settings [48]. While our results showed the ankle-worn AG accelerometer to be the most sensitive in distinguishing walking from non-ambulation and different walking speed categories, data fusion considering different axes could yield more sensitive outcomes, especially in individuals who have ankle-related joint problems. Although future work will entail analyzing raw acceleration data and the use of machine learning, the developed cut-points in this study are easy to implement and provide valuable information regarding which AG variables can be used to classify walking speed in people post stroke. 

Our study had some limitations, including the relatively small sample size that consisted of people with chronic stroke; therefore, our results are not generalizable to the overall stroke population. Another limitation was that the developed cut-points can only be used for AG accelerometers, especially since the AG software uses a proprietary amplitude threshold to determine a count. However, AG is one of the most used accelerometers to measure PA [10,11,12]. Although the AG proprietary threshold-based algorithm lacks adaptability and the use of other sensors (i.e., foot switcher) might be more sensitive in classifying different ambulation states, AG accelerometers have several benefits to clinical practices (i.e., could be worn on different body positions and measure PA for up to 6 weeks). The stroke participants also walked for 6 min, and it is unclear whether walking for such a prolonged time hindered their steady-state walking, especially when walking briskly. Moreover, individuals with stroke have a high variation in walking, especially people who walk slowly due to hemiparesis. This could decrease the robustness of the walking speed classifications, especially during shorter epoch lengths (e.g., 15 s). However, shorter epoch lengths might better reflect the variable nature of steady-state walking in daily living than longer epochs. We suggest that future work should also entail validating and applying the present AG cut-points in daily living in people post stroke. Such an analysis will ideally include an assessment of the activity counts variability to inform future calibration studies about reasonable epoch length in the stroke population.

## 5. Conclusions

Our results showed that the VM counts for the ankle-worn AG accelerometers to be most sensitive in differentiating between non-ambulatory and walking and in classifying different walking speeds in people post stroke. The resultant cut-points may be used to quantify PA behavior in people post stroke and to ascertain regular free-living walking speed in this population. In the clinical setting, the developed cut-points can be used as a complimentary measure to the clinical assessment of walking through clinical tests (e.g., 6 min and 10 m walk tests). The classification of walking speeds in daily living is of clinical interest post stroke due to the strong association between walking speeds and the risk of deteriorating health.

## Figures and Tables

**Table 1:** Non-ambulation and walking test conditions.

Task	Description and Instructions
**Non-ambulatory ^1^**	
Sitting (5 min)	The test leader conducted structured interviews related to demographics and personal factors while the participants were seated on a regular chair in front of a table.
Setting the table (3 min)	Participants sat on a chair with three kitchen crockery set placed in front of them and were asked to align all items as if they were setting the table for dinner. After completing this, participants returned the items back to the starting position and repeated the task.
Washing dishes (6 min)	Participants were equipped with a dish-cleaning brush and stood in front of a kitchen sink with a kitchen crockery set positioned on the left-hand side of the sink. The participants were asked to wash the dishes, dry the dishes with a cloth and position them on the right-hand side of the sink. This process was repeated, this time while moving the dishes from the right to the left-hand side.
**Walking ^2^**	
Self-selected walking speed (6 min)	Participants were asked to “walk at their normal comfortable walking speed for 6 min”.
Brisk walking speed (6 min)	Participants were asked to “walk as fast as they can in a safe manner, without running, for 6 min”.

^1^ The non-ambulatory activities are detailed in the work of Bezuidenhout et al. [34]. ^2^ The walking was performed on a track with 180 degree turns every 60 m, and participants were allowed to use a walking aid if needed.

**Table 2:** Participant characteristics.

Variables	People Post Stroke (*n* = 42)
Male sex, *n* (%)	19 (45)
Age (years), mean (SD)	63.4 (12.4)
Living alone, *n* (%)	15 (36)
Body mass index, mean (SD)	25.8 (3.5)
Mobility status, *n* (%)	
Unaided	30 (71)
Walking aid	12 (29)
Years since stroke, mean (SD)	2.4 (3.9)
Type of stroke, *n* (%)	
Ischemic	31 (74)
Hemorrhage	11 (26)
Affected side, *n* (%)	
Right	15 (36)
Left	27 (64)
NIHS stroke scale	2.1 (1.6)
MOCA score, mean (SD)	24.6 (3.5)
KATZ, *n* (%)	
Independent Primary ADL	35 (83)
Independent Instrumental ADL	20 (48)

Abbreviations: SD: standard deviation; *n*: number of participants; NIHSS: National Institutes of Health Stroke Scale; MOCA: Montreal Cognitive Assessment; and KATZ: Katz ADL Index Extended.

**Table 3:** Sample data used for the development of cut-points, including number of observations, median and confidence interval of ActiGraph counts in the non-ambulation and walking test conditions for the waist and ankle vertical and vector magnitude counts.

15 s Epoch						
	Waist			Ankle		
	*n*	Median (95% CI)		*n*	Median (95% CI)	
		Vertical	VM		Vertical	VM
Non-amb.	107	0 (7–39)	0 (15–70)	93	0 (7–22)	0 (15–39)
0.41–0.8 m/s	20	338 (283–441)	519 (495–790)	16	912 (724–1421)	1394 (1135–1890)
0.81–1.2 m/s	28	423 (341–477)	759 (621–847)	24	1898 (1444–2140)	2546 (2021–2774)
>1.2 m/s	22	555 (456–653)	1147 (976–1285)	15	3456 (2961–4354)	4045 (3479–4876)
**1 min Epoch**						
Non-amb.	107	17 (41–157)	44 (78–280)	93	14 (41–89)	37 (78–158)
0.41–0.8 m/s	20	1204 (997–1634)	1908 (1735–2932)	16	3496 (2593–5287)	5393 (4029–7064)
0.81–1.2 m/s	28	1628 (1265–1793)	2758 (2287–3181)	24	6777 (5265–8037)	9660 (7392–10,474)
>1.2 m/s	22	2099 (1580–2450)	4244 (3348–4839)	15	13,178 (10,023–16,569)	15,576 (11,774–18,619)

Abbreviations: Non-amb (non-ambulation), *n* (numbers), CI (confidence interval), m/s (meter per second), and VM (vector magnitude counts).

**Table 4:** ROC analysis for different ambulatory categories for 15 s and 1 min epochs.

	**15 s**								
		**Waist**				**Ankle**			
**Axis**	**Cat.**	**Sen.**	**Spec.**	**AUC**	**Cut-Point**	**Sen.**	**Spec.**	**AUC**	**Cut-Point**
**V**	Non-amb.	0.80	0.87	0.92	≤41	0.96	0.99	0.99	≤184
	0.41–0.8	0.70	0.57	0.60	42–372	0.75	0.83	0.79	185–1363
	0.81–1.2	0.58	0.74	0.66	373–502	0.79	0.91	0.91	1364–2851
**VM**									
	Non-amb.	0.95	0.93	0.97	≤140	0.96	0.99	0.99	≤401
	0.41–0.8	0.76	0.59	0.61	141–572	0.82	0.88	0.83	402–1862
	0.81–1.2	0.71	0.81	0.81	573–990	0.83	0.86	0.92	1863–3265
	**1 min**								
		**Waist**				**Ankle**			
**Axis**	**Cat.**	**Sen.**	**Spec.**	**AUC**	**Cut-Point**	**Sen.**	**Spec.**	**AUC**	**Cut-Point**
**V**	Non-amb.	0.77	0.92	0.91	≤270	0.92	0.99	0.98	≤570
	0.41–0.8	0.65	0.65	0.60	271–1458	0.76	0.76	0.76	571–4445
	0.81–1.2	0.53	0.79	0.63	1459–2055	0.73	0.89	0.84	4446–10,793
**VM**									
	Non-amb.	0.92	0.91	0.95	≤491	0.96	0.92	0.98	≤779
	0.41–0.8	0.72	0.66	0.61	492–2254	0.75	0.89	0.80	780–7200
	0.81–1.2	0.64	0.90	0.76	2255–4058	0.80	0.86	0.84	7201–12,487

Abbreviations: V (vertical counts), VM (vector magnitude counts), Cat (category), Sen (sensitivity), Spec (specificity), and Non-amb (non-ambulation). Speed categories are given in m/s.

**Table 5:** Developed walking speed cut-points for vector magnitude counts for waist- and ankle-worn ActiGraph.

	Waist VM Counts /15 s	Ankle VM Counts /15 s
Non-ambulation	≤140	≤401
0.41–0.8 m/s	141–572	402–1862
0.81–1.2 m/s	573–990	1863–3265
>1.2 m/s	≥991	≥3266

## Data Availability

With respect to the Swedish and EU personal data legislation (GDPR), the data are not freely accessible due to regulations regarding personal integrity in research, public access, and privacy.
